# Long Non-Coding RNAs in Gliomas: From Molecular Pathology to Diagnostic Biomarkers and Therapeutic Targets

**DOI:** 10.3390/ijms19092754

**Published:** 2018-09-13

**Authors:** Marek Vecera, Jiri Sana, Radim Lipina, Martin Smrcka, Ondrej Slaby

**Affiliations:** 1 Central European Institute of Technology, Masaryk University, 62500 Brno, Czech Republic; marek.vecera@ceitec.muni.cz (M.V.); sana.jiri@gmail.com (J.S.); 2 Department of Comprehensive Cancer Care, Masaryk Memorial Cancer Institute, Faculty of Medicine, Masaryk University, 62500 Brno, Czech Republic; 3 Department of Neurosurgery, University Hospital Ostrava, 70852 Ostrava, Czech Republic; radim.lipina@fno.cz; 4 Department of Neurosurgery, University Hospital Brno, Faculty of Medicine, Masaryk University, 62500 Brno, Czech Republic; Smrcka.Martin@fnbrno.cz

**Keywords:** glioma, glioblastoma, long non-coding RNA, biomarker, diagnosis, prognosis, molecular pathology

## Abstract

Gliomas are the most common malignancies of the central nervous system. Because of tumor localization and the biological behavior of tumor cells, gliomas are characterized by very poor prognosis. Despite significant efforts that have gone into glioma research in recent years, the therapeutic efficacy of available treatment options is still limited, and only a few clinically usable diagnostic biomarkers are available. More and more studies suggest non-coding RNAs to be promising diagnostic biomarkers and therapeutic targets in many cancers, including gliomas. One of the largest groups of these molecules is long non-coding RNAs (lncRNAs). LncRNAs show promising potential because of their unique tissue expression patterns and regulatory functions in cancer cells. Understanding the role of lncRNAs in gliomas may lead to discovery of the novel molecular mechanisms behind glioma biological features. It may also enable development of new solutions to overcome the greatest obstacles in therapy of glioma patients. In this review, we summarize the current knowledge about lncRNAs and their involvement in the molecular pathology of gliomas. A conclusion follows that these RNAs show great potential to serve as powerful diagnostic, prognostic, and predictive biomarkers as well as therapeutic targets.

## 1. Introduction

Gliomas are the most common malignancies of the central nervous system (CNS), accounting for almost 80% of all malignant neoplasia and about 30–40% of all primary tumors of the CNS [1]. The incidence of gliomas has been rising continually over the last decades, and the mortality curve still more or less follows a similar pattern to that of the age–incidence curve. The World Health Organization (WHO) assigned four grades of glioma malignancies, divided into two groups: low-grade (LGGs) and high-grade (HGGs) gliomas. LGGs include grade I and II gliomas, such as low-grade astrocytomas, low-grade oligodendrogliomas, and other mixed gliomas. HGGs include grade III tumors (anaplastic astrocytomas, anaplastic oligodendrogliomas, etc.) and glioblastoma multiforme (GBM, grade IV astrocytoma) [2].

Out of these, GBM is the most frequent primary malignant intracranial brain tumor. This heterogeneous, diffuse, and highly infiltrative disease is associated with unsatisfactory therapeutic outcomes and very poor prognosis [3,4,5]. The gold standard therapy consists of surgical resection, adjuvant concomitant chemoradiotherapy with temozolomide (TMZ), and in some cases subsequent TMZ as monotherapy [6,7]. Although median overall survival (OS) of patients under 70 is only 12 to 16 months from the diagnosis, OS ranges from 2.5 to 70 months [3,8]. Standard histopathological diagnostics is not able to both reflect these variabilities in survival and distinguish primary GBM subtype (~90%) arising de novo from secondary GBMs, slowly developed from anaplastic astrocytomas or LGGs [9]. It is thus important to study the molecular basis of GBMs and the impact of tumor molecular variability on the biological behavior of individual GBMs. Such knowledge will refine methods for both proper diagnosis and prediction of prognosis and therapeutic response [10]. Currently, the only significant molecular predictive marker of the response to the therapy with TMZ is the epigenetic silencing of the *MGMT* and *APNG* genes by the methylation of their promoters [11,12]. One positive prognostic molecular marker in GBM is the heterozygous mutation of the *IDH1* gene in codon 132. This marker has an occurrence frequency of about 12% and occurs almost exclusively in secondary GBM [13].

Since the biggest obstacles of cancer therapy, such as tumor therapy resistance and early recurrence, have not yet been overcome, the search for novel biomarkers of aggressive diseases such as GBM has steadily continued in recent years. In this search, certain molecular groups have elicited heightened interest. One such group is so-called non-coding RNAs (ncRNAs), that is, RNA transcripts of considerably varying length with no protein-coding potential. Although previously thought to be a transcriptional noise with no apparent function [14], they have been confirmed to serve structural and regulatory roles both in normal cellular physiology and in disease development and progression [15]. NcRNAs are divided into two distinct subtypes, with the length of the transcript being the main, albeit arbitrary, difference: small non-coding RNAs are shorter than 200 nucleotides (nts) while long non-coding RNAs (lncRNAs) are at least 200 nts long [16]. LncRNAs can be further divided into antisense lncRNAs, intronic lncRNAs, long intergenic non-coding RNAs (lincRNAs), promoter-associated lncRNAs, and untranslated region (UTR)-associated lncRNAs.

Structure-wise, lncRNAs are polyadenylated similarly to their messenger RNA (mRNA) counterparts, but they generally lack open reading frames [17]. They are predominantly present in the cell nucleus [18], expressed in a tissue-specific manner, and, unlike miRNAs and other small ncRNAs, poorly conserved between different species. LncRNAs have been found to be involved in transcriptional regulation, of which three main mechanisms have been observed: (1) acting in *cis*, by modulating expression of neighboring genes just by mere lncRNA transcription or through its RNA product; (2) acting in *trans*, by affecting the expression of distal genes via their RNA product [19]; and (3) regulating histone modification [20]. While over 50,000 human lncRNAs have been identified thus far, little is known about the physiological and aberrant functions of many individual lncRNAs [17,21]. This review focuses on the current knowledge of lncRNAs and their function as novel diagnostic, predictive, and prognostic biomarkers, and as the putative targets of future GBM treatment.

## 2. Role of Long Non-Coding RNAs in Molecular Pathology of Gliomas

Some studies combined computational and experimental methods to identify lncRNAs and their roles in tumor biology. Such a combinatorial approach could help discover not only the prognostic or predictive value of lncRNAs, but also their therapeutic potential. One such study came from Li et al., who analyzed 16 exon microarray datasets obtained from The Cancer Genome Atlas (TCGA), which included eight GBM and eight non-tumoral samples. Based on a constructed lncRNA–mRNA co-expression network, the authors predicted probable functions of several significantly dysregulated lncRNAs. They found 398 lncRNAs and 1995 mRNAs to be significantly dysregulated in GBM, and predicted the involvement of 98 lncRNAs in 30 pathways and 32 gene functions in connection to tumorigenesis, progression, and metastasis. They subsequently validated the identified set of GBM-specific lncRNAs by qRT-PCR in 30 GBM and 20 healthy brain tissues. Among them were predominantly unknown, upregulated lncRNAs ENST00000559148 and n410783, possibly involved in the cell cycle signaling pathway and in the mitogen-activated protein kinase (MAPK) signaling pathway, respectively. The downregulated lncRNAs included 5 lncRNAs whose adjacent sense-overlapped mRNAs were also downregulated and have been implicated in the regulation of cell adhesion and cell junction which may be related to the invasiveness of GBM [22].

Inspired by previously published data on lncRNAs in other types of cancer, some researchers looked for the same patterns in glioma. For example, Chun-Sheng Kang and his team studied HOX transcript antisense RNA (HOTAIR), one of the most extensively studied lncRNAs, and its involvement in GBM. They found that HOTAIR was overexpressed in HGG and that its upregulation was the predictor of poor survival [23]. In another study, they observed that HOTAIR promoted GBM cell cycle progression via the binding of its 5′ domain to PRC2 complex, which acts as histone methyltransferase and has a predominant component called EZH2. HOTAIR regulated the GBM cell cycle in a specific EZH2-dependent manner, implying that HOTAIR might be related to gene methylation and epigenetic silencing [24]. Moreover, HOTAIR expression negatively correlated with the expression of NLK, an important regulator of β-catenin and, therefore, the canonical Wnt pathway. The targeted downregulation of HOTAIR led to the inhibition of cell cycle progression and the invasiveness of GBM cells. In addition, GBM samples expressing high and low levels of HOTAIR had different MGMT status, suggesting HOTAIR is involved in DNA methylation [25]. HOTAIR was also shown to upregulate an 18-gene cell cycle-related mRNA network [26], and to participate in regulation of p53 signaling pathway [27] and angiogenesis through induction of vascular endothelial growth factor A (VEGFA) [28]. Due to this complexity of oncogenic functioning, we suggest HOTAIR to be a promising therapeutic target in GBM.

Two independent studies focused on lncRNA H19 and its involvement in the biology of glioma via the interaction with miRNAs. Zhao et al. found that H19 was significantly upregulated in 28 glioma tissues as well as in 5 human glioma cell lines compared to the adjacent normal tissues and normal human astrocytes (NHA), respectively. The knockdown of H19 caused reduced cell growth of both GBM cell lines U87MG and U251. In cell cycle, the knockdown caused the upregulation of p27 and the downregulation of cyclin D1 as well as a significant accumulation of cells in the G0/G1 phase. In particular, miR-140 was overexpressed after the downregulation of H19, and its expression level correlated inversely with the expression of H19. The forced upregulation of miR-140 led to a decrease in proliferation and cell cycle arrest of glioma cells while its downregulation had an opposite effect. Furthermore, H19 and the inhibitor of apoptosis-stimulating protein of p53 (iASPP) were the direct targets of miR-140. As expected, iASPP was overexpressed in glioma samples compared to paired normal tissues, and its expression correlated negatively with the expression of miR-140. H19 knockdown caused the expression level of iASPP to drop, and the targeted downregulation of iASPP inhibited cell growth. Therefore, the authors postulated H19 to be an oncogenic lncRNA that regulates iASPP through suppressing miR-140 [29]. Shi et al. found that H19 expression correlated with glioma grade and that the downregulation of H19 inhibited the invasiveness and migration of U87 and U251 cells. Since H19 was previously reported to serve as a precursor of miR-675-5p and miR-675-3p, the authors looked at the expression levels of these miRNAs in 185 glioma samples from Chinese Glioma Genome Atlas (CGGA). Mature miR-675 was indeed higher in HGG samples than in LGG ones. H19 knockdown reduced the expression level of miR-675, and the forced downregulation of miR-675 led to a decrease in cell invasiveness. Cadherin 13 (CDH13), a tumor suppressor in various types of cancer, was a direct target of miR-675. Therefore, H19 seems to function also via H19/miR-675/CDH13 axis by deriving miR-675, suggesting H19 is a candidate for the future therapy of gliomas [30]. H19 could promote proliferation and invasion of glioma cells also by sponging of miR-152 [31].

Clustering genes in the CGGA database according to the expression of homeobox protein HOXA11 antisense RNA (HOXA11-AS) and screening top 1000 positively or negatively correlated genes, Wang et al. discovered the prognostic value of HOXA11-AS and shed some light on its role in gliomas. Their analysis revealed that HOXA11-AS was highly associated with cell cycle progression. The stable overexpression of HOXA11-AS in glioma cell lines led to increased colony formation, S-phase rate, and the expression of proteins required for G1/S transition, such as cyclin D1, CDK4, cyclin E, and CDK2. In addition, the upregulation of HOXA11-AS decreased the expression of inhibitors of CDKs (p16, p21, and p27) and the expression of Rb, a known inhibitor of G1/S transition. Stable knockdown of HOXA11-AS via lentiviral particles had the exact opposite effect. While the injection of cells with overexpressed HOXA11-AS into nude mice resulted in increased tumor size, proliferation index (PCNA) and cell cycle factors (cyclin D1, p-Rb) decreased overall survival; the xenograft of cells with knocked-down HOXA11-AS again had the exactly opposite effect [32]. More recently, HOXA11-AS was further proved to promote glioma tumorigenesis through sponging of miR-140-5p [33]. 

Lv et al. observed higher expression of zinc finger E-box binding homeobox 1 antisense RNA (ZEB1-AS1) in three GBM cell lines than in an LGG cell line. Silencing of ZEB1-AS1 in glioma cells not only resulted in the inhibition of cell cycle, proliferation, migration, invasion, and increased apoptosis, but also in the upregulation of E-cadherin, an epithelial marker, and in the downregulation of mesenchymal marker N-cadherin. Knockdown of ZEB1-AS1 also decreased the expression levels of other molecules related to invasion and metastasis, such as Integrin-β1, MMP2, and MMP9. Hence, ZEB1-AS1 may affect cell migration and invasion via ZEB1-EMT (epithelial–mesenchymal transition) pathway in glioma [34].

Another study by Wang et al. found all four colorectal neoplasia differentially expressed (CRNDE) transcription variants to be upregulated in glioma cells compared to normal control cells. Its knockdown decreased the phosphorylation level of P70S6K, a gene directly downstream of mTOR, implying an association between the expression level of CRNDE and mTOR signaling pathway. Aberrant changes in this particular pathway caused increased tumor growth. Overexpression of CRNDE in glioma could promote cell proliferation and invasion in vitro and tumorigenesis in vivo, suggesting CRNDE’s role in glioma biology [35]. Together with H19 and HOTAIRM1, CRNDE was also found among lncRNAs upregulated in recurrent gliomas [36]. Recently, CRNDE was shown to promote glioma malignancy through activation of epidermal growth factor receptor (EGFR) signaling [37] and preventing miR-136-5p-mediated downregulation of Bcl-2 and Wnt2 [38]. 

Ma et al. characterized the role of lncRNA activated by transforming growth factor β (ATB) in the context of GBM cell lines U251 and A172, by studying the impact of its knockdown via transfection with sh-ATB plasmid. This knockdown resulted in the inhibition of cell viability, colony formation, colony size, migration, and invasiveness. They also found that ATB functioned as a ceRNA and directly interacted and inversely correlated with miR-200a. In an RNA immunoprecipitation experiment, miR-200a targeted ATB but did not degrade it. While the repression of miR-200a via its inhibitors restored the sh-ATB-induced inhibition of proliferation and colony formation in glioma cells, the combination of ATB knockdown and miR-200a overexpression resulted in the opposite outcome. Furthermore, overexpression of miR-200a markedly reduced both mRNA and protein expression levels of TGF-β2 via direct binding to its 3′ UTR. In the opposite scenario, the inhibition of miR-200a expression ended in TGF-β2 upregulation. While knockdown of ATB suppressed expression of TGF-β2, miR-200a inhibitors rescued TGF-β2 downregulation in ATB knockdown cells. Finally, the effect of ATB knockdown was also seen in xenograft nude mice, leading to decreased tumor growth [39].

According to another study, nuclear enriched abundant transcript 1 (NEAT1) was upregulated in 15 glioma tissues and 3 glioma cell lines compared to normal brain tissues. Knockdown of NEAT1 in vitro resulted in a decrease in invasiveness and migration and an increase in apoptosis rate, while in vivo in reduced tumor growth. The knockdown was negatively related to miR-449b-5p and its direct functional target. Similarly, this miRNA also targeted notorious oncogene c-Met. Based on a series of experiments, the authors concluded that NEAT1 acted as an oncogenic lncRNA via the miR-449b-5p/c-Met axis [40] and via modulation of transcription factor SOX2 targeted by miR-132 [41]. Metastasis associated lung adenocarcinoma transcript 1 (MALAT1), a strong positive regulator of invasion associated with cell migration, was regulated by WIF1 expression via the WNT5A/p38-MAPK/Ca^2+^ non-canonical Wnt signaling axis. Although a better understanding of this particular pathway could reveal potential therapeutic targets in glioma, thr authors of this study concluded that MALAT1 is not a good candidate due to its relatively high basal level of expression in normal brain tissue [42]. Knockdown of SPRY4 intronic transcript 1 (SPRY4-IT1), another upregulated lncRNA in glioma, was linked to reduced proliferation, migration, and EMT [43].

Studying the role of taurine upregulated 1 (TUG1) in GBM, Cai et al. proposed that, together with miR-299, TUG1 is a part of a repression feedback loop. Overexpression of miR-299 significantly suppressed spheroid-based tumor-induced formation of blood vessels in vitro and tumor growth in vivo, while knockdown of TUG1 decreased the expression level of VEGFA, a molecular switch of tumor angiogenesis. VEGFA was also directly targeted by miR-299, suggesting that this miRNA might be involved in the TUG1-mediated regulation of angiogenesis. This regulation process, in turn, would make TUG1 a potential target of future GBM therapy [44]. Another—rather conflicting—study by Li et al. found TUG1 to be gradually more downregulated across the glioma grades. They observed that in vitro overexpression of TUG1 could promote cell apoptosis while inhibition via siRNA-mediated silencing increased proliferation. Western blot analysis revealed that TUG1 overexpression decreased the expression level of anti-apoptotic protein Bcl-2. Authors of these TUG1 studies focused on different biological features of glioblastoma (angiogenesis versus proliferation/apoptosis). From this perspective, results are not contradictory. However, dysregulation of TUG1 expression in gliomas observed in these studies were not in agreement, which could be partly explained by low number of cases in the study by Cai et al. (*n* = 5 of each glioma grade) [36]. Downregulation of TUG1 observed by Li et al. was described in 120 paired specimens of tumor and respective non-tumor tissue [45]. Another lncRNA described to be involved in regulation of angiogenesis is X-inactive specific transcript (XIST). Knockdown of XIST increases blood–tumor barrier permeability and inhibits glioma angiogenesis by targeting miR-13 [46].

Maternally expressed gene 3 (MEG3), a confirmed tumor suppressor lncRNA, plays a role in cell proliferation, by interacting with cyclic AMP, p53, MDM2 and GDF15. Its expression is epigenetically controlled, and aberrant CpG methylation has been reported in several types of cancer. Wang et al. studied MEG3 in 17 glioma samples (WHO grade 0–III) and the adjacent healthy tissues as well as stable cell lines U251 and U87MG. Its expression was reduced in 82% of gliomas as compared to the healthy tissues. Stable transfection of pcDNA-MEG3 into cell lines reduced their proliferation and increased apoptosis. RNA immunoprecipitation (RIP) assay with antibody against p53 and RNA pull-down revealed a significant enrichment of MEG3 and p53, respectively. In U251 cells, elevated expression of MEG3 increased p53 activity and mRNA levels of caspase 8/3 and TP53 [47]. These results suggest an association between MEG3 with p53. Later studies confirmed involvement of MEG3 in regulation of Wnt/beta-catenin signaling pathway [48] and through targeting of miR-93 also its ability to inactivate PI3K/AKT pathway [49]. Another lncRNA observed to be positively affecting progression of glioma via activating Wnt/beta-catenin signaling is differentiation antagonizing non-protein coding RNA (DANCR) [50].

Tumor suppressor candidate 7 (TUSC7), another tumor suppressor lncRNA, was downregulated in 39 glioma samples compared to 17 adjacent healthy brain tissues. Similar results were observed in stable cell lines U251 and U87MG relative to NHA cells. The expression level of TUSC7 negatively correlated with the histological grades of gliomas and was a prognostic biomarker in glioma patients. Stable overexpression of TUSC7 in both U251 and U87MG led to the inhibition of cell viability, migration, and invasion and an increase in apoptosis, as compared to NHA. Bioinformatics analysis found a miR-23b binding site in the TUSC7 transcript and predicted TUSC7 to be a target gene of miR-23b. Moreover, the expression level of TUSC7 negatively correlated with that of miR-23b. While agomiR-23b downregulated TUSC7 expression, antagomiR-23b upregulated it. The dual-luciferase reporter assay confirmed the direct binding of miR-23b to TUSC7. An RNA pull-down experiment showed a significant enrichment of TUSC7 with biotinylated miR-23b, and vice versa. A RIP experiment with Ago2 antibody confirmed that these two RNAs coprecipitated. Stable overexpression of TUSC7 markedly reduced miR-23b expression in both cell lines. While the downregulation of miR-23b impeded viability, migration, and invasion as well as increased apoptosis, its upregulation could partly reverse the inhibitory effect of TUSC7, substantially preventing apoptosis [51]. Similar results were reported for tumor suppressor lncRNA CASC2 and its reciprocal partner, miR-21 [52].

Apart from discovering the predictive value of RP11-838N2.4, Liu et al. also found that, after the treatment of cells with TMZ, in vitro stable transfection with pcDNA-RP11-838N2.4 (pcDNA-RP) plasmid enhanced the sensitivity of cells to TMZ and increased apoptosis. Xenograft studies also confirmed that overexpression of RP11-838N2.4 enhances cytotoxicity of TMZ in U87TR in vivo. Out of all candidate miRNAs validated to be involved in resistance against TMZ in glioma, miR-10a was the most downregulated in U87TR transfected with pcDNA-RP. It was also significantly downregulated in U251TR transfected with pcDNA-RP and upregulated in both resistant cell lines relative to their nonresistant counterparts. Dual-luciferase reporter assay proved that miR-10a was directly binding to RP11-838N2.4, while RIP assay with Ago-2 antibody proved that both RNAs coprecipitated together. In addition, miR-10a was upregulated in recurrent GBM compared to primary GBM. Another target of miR-10a, EphA8, was downregulated when miR-10a was upregulated. A series of experiments proved that RP11-838N2.4 possessed the ability to enhance TMZ cytotoxicity in GBM via EphA8-dependent manner. Finally, RP11-838N2.4 was able to attenuate TGF-β signaling, whose role in TMZ resistance had already been known. However, this mechanism works independently of miR-10a [53]. 

More recently, well described tumor suppressive lncRNA, GAS5, was described to suppress malignancy of glioma stem cells via a miR-196a-5p/FOXO1 feedback loop [54] and proliferation, migration, and invasion of glioma cells by negative regulation of miR-18a-5p [55]. LncRNA known from pathogenesis of bladder cancer called UCA1 has been shown to modulate glioblastoma-associated stromal cells-mediated glycolysis and invasion of glioma cells [56]. Well known oncogenic and MYC-associated lncRNA PVT1 facilitates tumorigenesis and progression of glioma via regulation of miR-128-3p/GREM1 axis and BMP signaling pathway [57].

**Figure 1:**
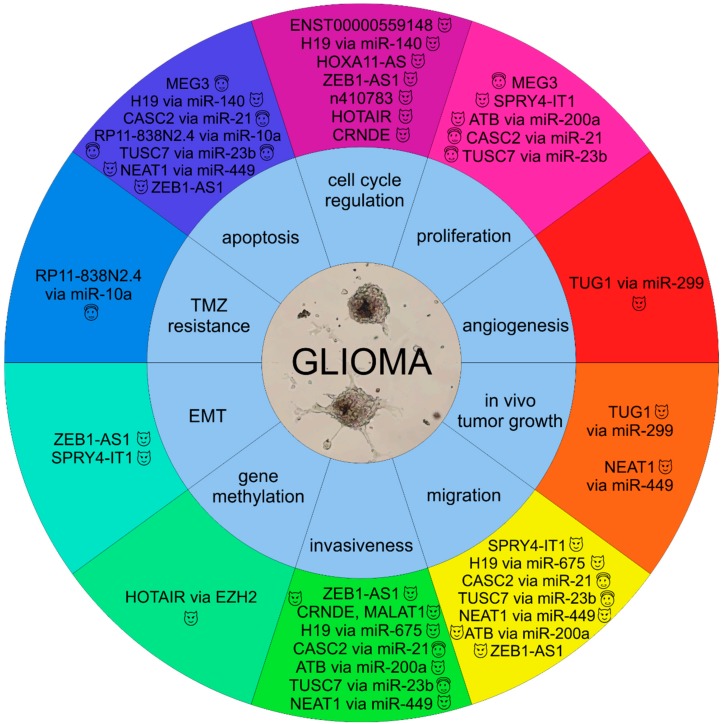
Involvement of selected lncRNAs in the molecular pathology of gliomas (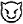, oncogenic lncRNAs; 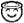, tumor suppressor lncRNAs).

Figure 1 summarizes the involvement of selected lncRNAs in the molecular pathogenesis of gliomas.

## 3. Long Non-Coding RNAs as Diagnostic Biomarkers in Gliomas

Many studies have used microarrays and RNA for the comparative molecular lncRNA profiling of tumor and non-tumor tissues. Several studies aimed to identify lncRNAs that could serve as diagnostic markers of particular subtypes of glioma or, more specifically, GBM. One such study employed two large cohorts of glioma gene expression data from Gene Expression Omnibus (GEO), GSE16011 (training set) and GSE4290 (test set), which contained both WHO grade II–IV glioma and non-tumoral brain samples. The authors identified 102 lncRNAs dysregulated between all glioma samples and normal tissues, 127 lncRNAs with differential expression in high-grade astrocytomas (HGAs), and 103 lncRNAs dysregulated between low-grade astrocytomas (LGAs) and normal tissues. Additionally, 23 lncRNAs were downregulated while 10 were upregulated in HGAs compared to LGAs. In total, 10 lncRNAs followed a grade-dependent expression pattern in astrocytomas from the training set, of which CRNDE and HOTAIRM1 were upregulated with ascending malignancy grades, while MEG3, C21orf131-B, and PAR5 were downregulated. Later, validation excluded four lncRNAs in the test set, including all three downregulated lncRNAs. Using the exact same procedure in oligodendrogliomas, 21 lncRNAs had a significant differential expression between high-grade (HGOs) and low-grade oligodendrogliomas (LGOs). In comparison with normal tissues, 106 dysregulated lncRNAs were identified in HGOs and 99 dysregulated lncRNAs in LGOs. In both training and test sets, LncRNAs LOC286002, C21orf131-A, and C21orf131-B were tightly associated with the malignancy scale of oligodendrogliomas. Finally, this study identified, in 265 samples, 23 lncRNAs (including NEAT1 and DLX6-AS) which could differentiate between astrocytomas and oligodendrogliomas, with 87.7% accuracy [58].

Another study used profiles of 1970 lncRNAs from the Rembrandt dataset, consisting of 475 samples of astrocytic, oligodendroglial, or mixed origin and normal brain tissues. Bioinformatics analysis revealed three lncRNA-based subtypes (LncR1, LncR2, and LncR3), to which three out of four identified gene signatures corresponded. The same results were obtained in a validation set, GSE16011. While LncR1 showed the worst prognosis and had most EGFR amplifications, LncR3 had the longest overall survival and most IDH1 mutations and 1p/19q LOH. LncR2 had an intermediate clinical outcome. Furthermore, tumors from LncR1 were of astroglial origin, LncR2 subtype possessed characteristics of neuronal cell lineage, and LncR3 was enriched with the oligodendrocytic signature [59].

To achieve reliable and reproducible data from qRT-PCR, Kraus et al. aimed to find stably expressed lncRNA normalizers. Using the human LncProfiler qPCR assay, a related lncRNA database of annotated lncRNAs, and the NormFinder algorithm, they analyzed 90 lncRNAs in a cohort of 30 gliomas (5 diffuse astrocytomas, 5 anaplastic astrocytomas, 15 GBM, and 5 normal white matter brain samples), in which they identified 24 lncRNAs suitable as normalizers. Out of these, the authors selected seven lncRNAs which fulfilled all chosen criteria to be considered as stably expressed in all tumor samples. Among them, the five most stable lncRNAs were Zfhx2as, SNHG4, HOXA6as, HUC1&2, and ncR-uPAR. By adding expression data from normal white matter, four lncRNAs remained in the selection, including HOXA6as, HUC1&2, Zfhx2as, and BC200—the authors regarded them as universal normalizers [60].

In addition to using glioma tissues samples, another team of researchers used peripheral blood samples and investigated diagnostic potential of circulating lncRNAs. The authors found 80 differentially expressed lncRNAs in a cohort of 28 tumor tissue samples compared to 10 normal healthy brain tissues. After several rounds of selection, miR210HG was chosen for further research, based on the observed changes and its ability to bind to BMP1. Stable miR210HG could be detected in all serum samples collected from 10 glioma patients and 10 healthy volunteers. Expression levels of miR210HG were generally higher in sera from glioma patients and also higher in a high-risk group (WHO III or IV). Finally, ROC analysis revealed that, as a biomarker of glioma, miR210HG had the sensitivity of 86.21% and the specificity of 72.41% [61]. Table 1 summarizes potential diagnostic lncRNAs in gliomas.

## 4. Long Non-Coding RNAs as Prognostic and Predictive Biomarkers in Gliomas

Various experimental and computational approaches have been devised to identify lncRNAs and their functions in tumor biology, such as the aforementioned microarray analyses and RNA-seq [62] and the construction of functional lncRNA-mediated networks [22,63]. Several recent studies have employed these two approaches to find putative prognostic or predictive markers and correlated them with tumor progression and survival. Zhang et al. used a lncRNA-mining approach to perform lncRNA expression profiling in 213 GBM tumors from TCGA, and validated their results in two independent testing sets of 68 and 101 specimens. They determined a six-lncRNA signature that significantly correlated with the patients’ OS. Among these six lncRNAs were previously described lncRNAs PART1, MIAT, and GAS5, which, together with another two, were associated with lengthened survival; higher expression of one lncRNA, KIAA0495, was associated with decreased survival. The prognostic value of this lncRNA set was independent of both age and MGMT promoter methylation status [64].

Another study by the same team focused on lncRNA expression profile changes in gliomas associated with IDH1(R132) mutation. The authors applied the lncRNA expression profile mining method on three datasets obtained from GEO database and identified several dysregulated lncRNAs between IDH1-wildtype and IDH1-mutant GBMs. Among the most downregulated lncRNAs in IDH1-mutant GBMs were KIAA0495 and HOTAIRM1. The former was previously described in oligodendrogliomas as a tumor suppressor and a modulator of p53-dependent regulation of anti-apoptotic genes, while HOTAIRM1 was suggested to be involved in the regulation of leukemia cell cycle progression [65]. Zhi et al. found 59 lncRNAs that were differentially expressed in three tumor samples (WHO grade II–IV) compared to three normal brain samples and met chosen criteria. These lncRNAs were then validated by qRT-PCR in 130 astrocytoma samples and 60 normal adjacent tissues. The authors subsequently determined a set of seven lncRNAs which could significantly differentiate between tumor and healthy tissues, and they established a possible link between advanced clinical stages of astrocytomas and higher expression levels of lncRNAs ENST00000545440 and NR_002809. Moreover, high expression of NR_002809 and low expression of BC002811 and XLOC_010967 were significantly associated with shortened survival [66]. Similar results were obtained for lncRNA HOXA11-AS by Wang et al. [32].

More recently, Wang et al. analyzed three datasets containing lncRNA expression profiles of 183 anaplastic astrocytomas. Univariate Cox analysis and the risk score method revealed a potential prognostic value in a four-lncRNA signature (AGAP2-AS1, TPT1-AS1, LINC01198, and MIR155HG), because it could significantly distinguish between the low-risk group of patients and the high-risk one. With more prominent expression in the low-risk group, TPT1-AS1 was postulated to have a protective role; the other three lncRNAs were expressed more in the high-risk group. Unlike the above-mentioned studies, this study additionally comprised in vitro experiments, which showed that in vitro siRNA-mediated knockdown of AGAP2-AS1 in GBM cell lines L215N229 and U87MG inhibited cell proliferation while the downregulation of AGAP2-AS1 suppressed invasiveness and increased the rate of apoptosis [67].

Some researchers tried to transfer results published for other cancers into glioma research. Hu et al. found that lncRNA AB073614 was overexpressed in 65 glioma samples, but not in 13 non-tumor controls. It was also upregulated in HGGs relative to LGGs. Kaplan–Meier analysis and a log-rank test revealed that AB073614’s expression correlated with the overall survival of the patients and thus has potential as a negative prognostic biomarker for glioma [68]. Jing et al. obtained similar results for lncRNA CRNDE in 164 glioma and adjacent non-tumor tissues. They observed overexpressed CRNDE in tumor tissues, which was associated with higher WHO grade, recurrence, and tumor proportions. Its high expression was an independent prognostic marker of poor prognosis for patients with glioma [69]. In another study, relative expression of SPRY4-IT1 was higher in 163 glioma samples than the adjacent non-tumor tissues, and it correlated with both WHO grade and tumor size. Thus, the authors applied Kaplan–Meier analysis, a log-rank test, and univariate and multivariate analyses, which confirmed SPRY4-IT1 had potential as an independent negative prognostic marker related to overall survival in glioma [70]. Following several studies suggesting ZEB1-AS1 expression was associated with poor outcomes of patients with hepatocellular carcinoma and esophageal squamous cell carcinoma, Lv et al. found that this lncRNA was significantly upregulated in 82 glioma samples compared to 13 healthy controls. In addition, higher expression of ZEB1-AS1 correlated with tumor grade and was independent negative prognostic factor for overall survival [34]. Similar results were reported for MALAT1 [71]. In none of these studies, lncRNA expression was associated with gender, age, or tumor location [34,68,69,70,71].

A team led by Xiao and Li used a multi-step computational approach to identify prognostic biomarkers in GBM, focusing on the cross-talk between so-called competitive endogenous RNAs (ceRNAs). CeRNAs compete for shared miRNAs as so-called miRNA sponges and include lncRNAs and mRNAs. By utilizing genome-wide lncRNA and mRNA expression profiles of 422 GBM samples from TCGA and identified miRNA-target interactions, the authors constructed a functional GBM lncRNA-mediated ceRNA network (LMCN), which consisted of 393 lncRNAs, 4176 mRNAs, and 16,860 ceRNA interactions. According to this study, lncRNAs in the LMNC were more likely to serve as central regulators than mRNAs based on the ceRNA regulatory behavior, and the whole LMCN was similar to many biological networks. MCM3AP-AS, a lncRNA mapped to the LMCN, affected survival and was able to divide the same cohort of 422 patients into two different risk groups. Therefore, the authors regarded MCM3AP-AS as a potential protective and prognostic factor for survival in GBM. This study also found that MCM3AP-AS competed with another lncRNA (MIR17HG) and three mRNAs (MATR3, CCHC14, and XPO1). Together, they comprise a five-ceRNA module that could also divide patients from the same GBM dataset and from another independent cohort into a high-risk and a low-risk group, with even higher accuracy [63]. Table 2 summarizes potential prognostic lncRNAs in gliomas.

Other researchers searched for novel predictive biomarkers for glioma to be used in the conventional therapy of GBM. Based on experimental microarray data and qRT-PCR, RP11-838N2.4, a novel transcript of lncRNA GAPLINC, was recognized as downregulated in previously established TMZ-resistant stable cell lines U87TR and U251TR compared to nonresistant cells. Furthermore, the downregulation of RP11-838N2.4 positively correlated with both TMZ resistance and poor patient survival [41]. Wang et al. used a network-based co-expression algorithm called weighted gene correlation network analysis (WGCNA) to compare samples without irradiation with those exposed to radiation (2 and 6 h of post-irradiation). They found 43 interaction pairs, which contained 34 genes and 7 lncRNAs, triggered by irradiation. Out of these, another algorithm identified 20 predictors (16 genes and 4 lncRNAs) for developing a radiosensitivity model. One lncRNA, TP53TG1, was previously associated with both p53 expression and radiosensitivity. The model based on those 20 predictors accurately divided patients into two groups based on overall survival in two independent GBM cohorts. Thus, the model could serve as a predictive biomarker to identify patients who should respond to radiotherapy [72]. More recently, MALAT1 was described to decrease sensitivity of glioblastoma cell lines to TMZ [73]. Jia et al. has shown that silencing of H19 decreases chemoresistance of glioma cells to TMZ by suppressing epithelial–mesenchymal transition via the Wnt/beta-catenin pathway [74]. On the other hand, upregulation of CASC2 sensitized glioma to TMZ through autophagy inhibition by sponging miR-193a-5p and regulation of mTOR expression [75].

## 5. Conclusions

Despite improvements in treatment for high-grade gliomas, prognosis remains poor and presents a serious clinical problem. Finding both new therapeutic targets and new biomarkers enabling stratification of patients according to tumor biology is crucial and can be achieved by employing state-of-the-art technologies such as high-throughput genomic and transcriptomic investigations and computational bioinformatics analyses. These approaches help study novel molecular entities and their relationships with the biological behavior of tumor cells and, in turn, clinical outcomes.

It is because of their unique, compartmentalized expression and function in a wide range of regulatory processes in cancer cells that lncRNAs present promising candidates as biomarkers and therapeutic targets. Several lncRNAs have recently been identified as tumor suppressors or oncogenes in gliomas, and several functional categories have been discovered, such as lncRNAs producing miRNAs or lncRNAs acting as ceRNAs/miRNA sponges. The involvement of lncRNAs has been observed in various biological processes, such as epigenetic silencing, cell cycle progression, angiogenesis, and ionizing radiation resistance. Clinical potential of these molecules is also supported by many studies suggesting lncRNAs to be powerful diagnostic, prognostic, and predictive biomarkers in gliomas. To fully comprehend their therapeutic and diagnostic value, however, and to draw further conclusions, we need deeper understanding of lncRNAs in molecular pathology of gliomas.

## Figures and Tables

**Table 1:** LncRNAs with diagnostic potential in gliomas.

LncRNA	Ensembl ID	Chromosome	Length (bp)	Tumor	References
HOXA6as	ENSG00000254369	7	25,952	astrocytoma	[60]
HUC1&2	-	11	400	astrocytoma	[60]
Zfhx2as	ENSG00000157306	14	49,019	astrocytoma	[60]
BC200	ENSG00000236824	2	200	astrocytoma	[60]
CRNDE	ENSG00000245694	16	84,001	astrocytoma	[58]
HOTAIRM1	ENSG00000233429	7	4619	astrocytoma	[58]
LOC286002	-	7	32,488	oligodendroglioma	[58]
C21orf131	ENSG00000224924	21	60,627	oligodendroglioma	[58]
NEAT1	ENSG00000245532	11	22,767	both	[58]
DLX6-AS	ENSG00000231764	7	58,925	both	[58]
miR210HG	ENSG00000247095	11	2801	both	[61]

**Table 2:** LncRNAs with prognostic potential in gliomas.

LncRNA	Ensembl ID	Chromosome	Length (bp)	Tumor	Effect on Prognosis	References
MCM3AP-AS	ENSG00000215424	21	30,174	GBM	positive	[63]
PART1	ENSG00000152931	5	59,945	GBM	positive	[64]
MIAT	ENSG00000225783	22	30,051	GBM	positive	[64]
GAS5	ENSG00000234741	1	4983	GBM	positive	[64]
RP11-838N2.4	ENSG00000266835	18	12,729	GBM	positive	[53]
KIAA0495	ENSG00000227372	1	11,773	GBM	negative	[64]
HOTAIR	ENSG00000228630	12	12,649	GBM	negative	[23]
BC002811	-	-	-	astrocytoma	positive	[66]
XLOC_010967	-	-	-	astrocytoma	positive	[66]
ENST00000545440	ENSG00000255717	11	3927	astrocytoma	negative	[66]
NR_002809	ENSG00000212694	12	8640	astrocytoma	negative	[66]
TPT1-AS1	ENSG00000170919	13	52,069	glioma	positive	[67]
TUSC7	ENSG00000243197	3	14,347	glioma	positive	[51]
HOXA11-AS	ENSG00000240990	7	4776	glioma	negative	[32]
AGAP2-AS1	ENSG00000255737	12	2117	glioma	negative	[67]
LINC01198	ENSG00000231817	13	31,372	glioma	negative	[67]
MIR155HG	ENSG00000234883	21	13,260	glioma	negative	[67]
AB073614	-	3	1910	glioma	negative	[68]
CRNDE	ENSG00000245694	16	84,001	glioma	negative	[69]
SPRY4-IT1	ENSG00000281881	5	703	glioma	negative	[70]
ZEB1-AS1	ENSG00000237036	10	114,170	glioma	negative	[34]
MALAT1	ENSG00000251562	11	8755	glioma	negative	[71]
